# Is it appropriate to conduct a disproportionality analysis using a spontaneous reporting database to investigate whether drug-related adverse events are dose-dependent?

**DOI:** 10.3389/fphar.2025.1563524

**Published:** 2025-08-29

**Authors:** Yoshihiro Noguchi, Tomoya Tachi, Tomoaki Yoshimura

**Affiliations:** ^1^ Laboratory of Clinical Pharmacy, Gifu Pharmaceutical University, Gifu, Japan; ^2^ Department of Clinical Pharmacy, Graduate School of Pharmaceutical Sciences, Nagoya City University, Nagoya, Aichi, Japan

**Keywords:** disproportionality analysis, spontaneous reporting database, drug-related adverse events, dose-dependent, signals of disproportionate reporting (SDR)

## 1 Introduction

The inherent limitations of key clinical studies on which marketing authorization is based leave uncertainty regarding rare or delayed adverse drug reactions (ADRs). To address these uncertainties, drug safety surveillance activities are undertaken to characterize the safety profile of drugs in real-world situations. One of the information sources underlying post-marketing drug safety surveillance is the spontaneous report database, in which ADRs suspected by healthcare professionals and patients are collected primarily through spontaneous reporting. The results of the spontaneous report database analysis are used as the basis for various regulatory responses that can lead to the withdrawal or discontinuation of a drug from the market, issuance of urgent safety information, and revision of the package insert (Noguchi et al., 2021a; Noguchi and Yoshimura, 2024).

**FIGURE 1:**
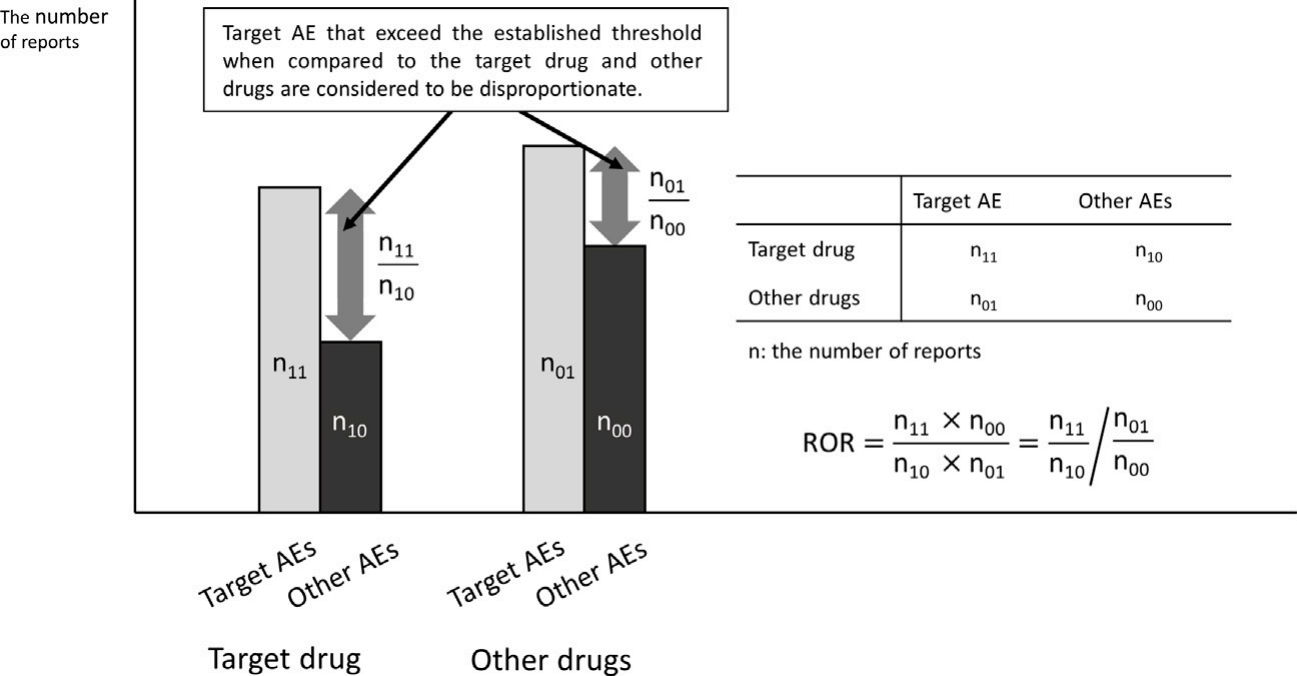
Principles of disproportionate measures (ROR, reporting odds ratio).

Major spontaneous reporting databases include VigiBase by the World Health Organization-Uppsala Monitoring Centre (WHO-UMC, 2025), Food and Drug Administration (FDA) Adverse Event Reporting System (FAERS) by the United States (US Food and Drug Administration, 2025), EudraVigilance by the European Medicines Agency (EMA) (European Medicines Agency, 2025), and Japanese Adverse Drug Event Report database (JADER) by the Pharmaceuticals and Medical Devices Agency (PMDA) in Japan (Pharmaceuticals and Medical Devices Agency, 2021). These databases only register cases in which ADRs have developed and do not include cases in which no ADRs occurred. Therefore, the incidence of ADRs cannot be calculated, and the search for ADRs generally uses Signals of Disproportionate Reporting (SDR) by disproportionality analysis (Figure 1). The well-known indicators include the Reporting Odds Ratio (ROR) (Rothman et al., 2004) and the Proportional Reporting Ratio (PRR) (Rothman et al., 2004; Evans et al., 2001), which are frequentist statistical methods, and the Bayesian Confidence Propagation Neural Network (BCPNN) (Bate et al., 1998) and Empirical Bayesian Geometric Mean (EBGM) (DuMouchel, 1999), which are Bayesian statistical methods.

In recent years, improvements in computer processing power have made it easier for laboratories to handle large data sets, making it possible for those not affiliated with regulatory agencies or pharmaceutical companies to conduct analyses of spontaneous reporting databases (Urushihara, 2019). A search of PubMed for articles on disproportionality analysis reported an increase from 40 in 2017 to 180 in 2021 (Khouri et al., 2021a). However, researchers often forget that the results of disproportionality analysis are merely clinical hypotheses. Over 2/3 of the authors reported overestimating or misinterpreting the findings (Mouffak et al., 2021). The increase in the number of these inappropriately reported papers has become a social problem, and reporting guidelines for disproportionality analysis (Reporting of A Disproportionality analysis for drUg Safety signal detection using spontaneously reported adverse events in PharmacoVigilance; READUS-PV) have been published (Fusaroli et al., 2024). On the other hand, few studies summarize important points to note regarding methodology.

ADRs can be dose-dependent or mechanism-of-action-based. Therefore, ADR searches using spontaneous report databases (Alexandre et al., 2024; Hatano et al., 2023; Gu and Samarneh, 2025) have also produced an increasing number of reports on drug dose dependence. Although these studies have the potential to provide significant clinical findings, there are cases where analyses have been conducted beyond the research limitations of the spontaneous report databases.

This paper highlights five key points for evaluating dose dependence using spontaneous report databases.

## 2 Five key points to understand

First, because spontaneous reporting databases are susceptible to various reporting biases, there is no guarantee that the reported cases adequately represent all patients treated with the drug. When investigating the association between ADR and dose-dependence, many researchers focus on the number of reports each dose group. If the number of ADR reports increases with increasing dose, an association between ADR and dose dependence may be established. However, the number of reports in the spontaneous reporting database does not necessarily reflect the incidence rates of ADRs. The spontaneous reporting database does not contain data that would allow us to determine the total number of patients using the drug. In other words, if the total number of patients using the drug in the low-dose group is small, the number of reported registered cases will naturally be small, even though the actual incidence rate is not different from that in the high-dose patient group.

**FIGURE 2:**
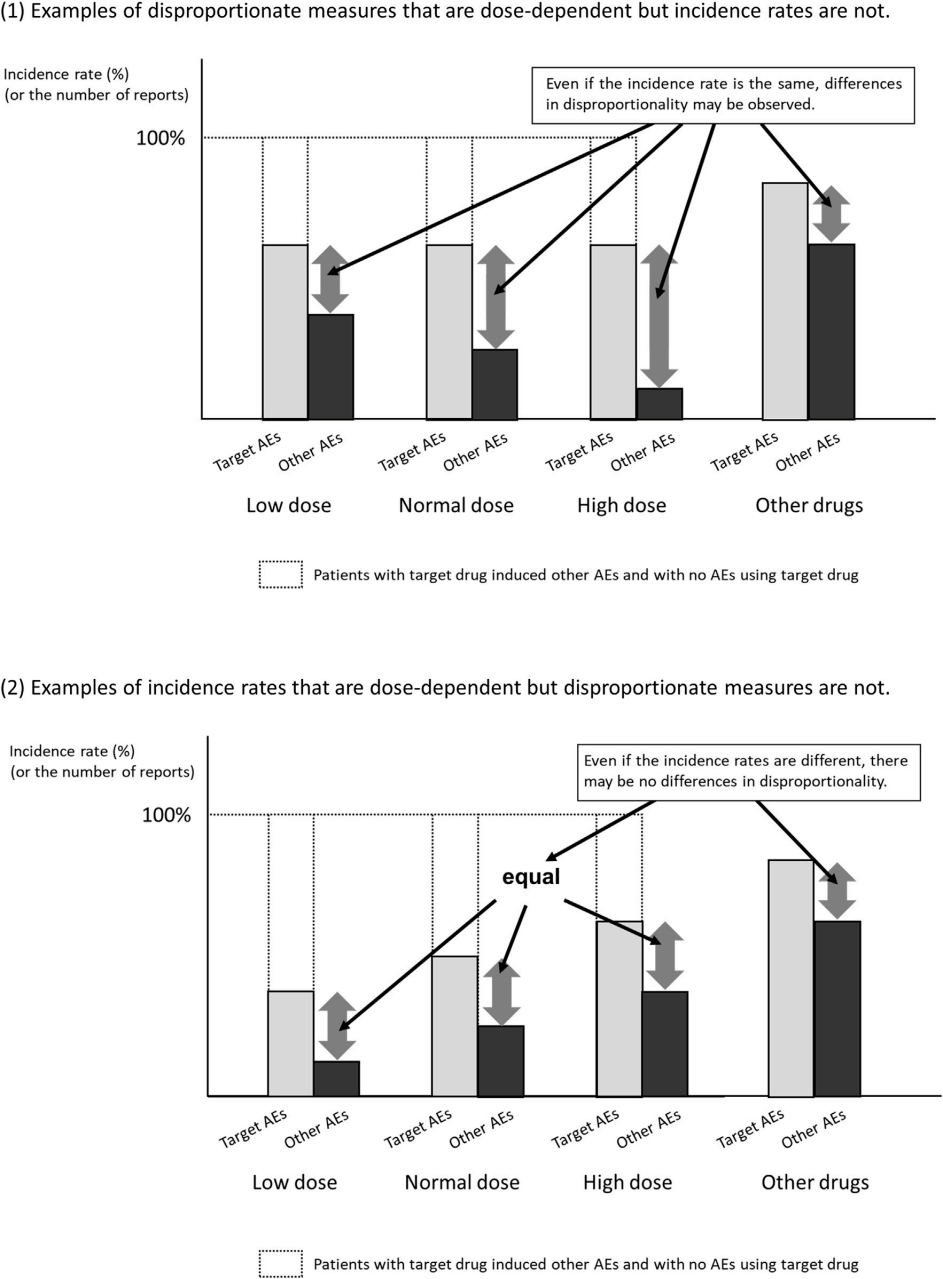
Examples of association between disproportionality and dose-specific incidence rates.

Second, the SDR score is not necessarily related to the magnitude of the risk of developing ADRs. The control group in the disproportionality analysis using the spontaneous reporting database is not the group that did not develop ADRs, but the group that developed ADRs other than the target ADRs. As shown in Figure 2, when ADRs other than the target ADR are inversely correlated with dose, the results may be as if the SDR score of the target ADR is dose-dependent, even though the incidence of the target ADR is the same. For example, when infections are reported as ADR of antibiotics, many of them are due to insufficient antibiotic doses. Therefore, even if there is a dose-dependence in disproportionality, it is unclear whether there is a dose-dependence in the risk of developing ADR.

It is true that there are long-standing reports of similarity between SDR scores and the results of randomized trials (Beau-Lejdstrom et al., 2019) and meta-analyses (Khouri et al., 2021b), but there are too few examples to make a positive observation. Recent studies have reported that the ROR score tends to be inflated out of the SDR score when the number of reports is small (Fusaroli et al., 2024; Noguchi et al., 2021b). In addition, as shown in Figure 2, even if the number of reports shows dose-dependent, it is entirely possible that the disproportionate measures don’t demonstrate dose-dependence. That is, we should probably follow the current recommendation that disproportionate measures not be used as valid substitutes for risk estimates. Therefore, even if there is some association between SDR score and drug dose, one should be cautious in making the clinical hypothesis that the risk of developing ADRs is dose-dependent.

Third, it is known that there is a lot of missing data in spontaneous reporting databases. For example, Alexandre et al. used the VigiBase to examine the association between ibrutinib dosing regimens and reports of ibrutinib-related atrial fibrillation (IRAF) in patients with chronic B-cell malignancies to determine whether IRAF is dose-dependent (Alexandre et al., 2024). The VigiBase covers more than 35 million reports worldwide and includes management details, patient demographics, ADR occurrence data, appropriate drug information, and dosing frequency. According to their findings, the VigiBase had 33,623 ibrutinib-related reports in adults. Unfortunately, almost half lacked information on daily dosing, leaving only 18,498 reports available for analysis (Alexandre et al., 2024). Some researchers believe that excluding cases where doses were not recorded will still provide unbiased estimates; however, this is a major misconception. One should not assume that the patient population used in the analysis adequately reflects the population distribution of ADR incidence. Spontaneous reporting databases are subject to various reporting biases; therefore, the dose distribution of cases where doses were recorded does not necessarily match that of cases where doses were not recorded.

Fourth, it is important to consider the background behind the existence of different dosing data. For example, (Alexandre et al., 2024), ibrutinib is administered orally once daily at either 420 mg or 560 mg, depending on the indication. Dose reductions are determined based on the frequency of Grade 3 adverse events, with more frequent events requiring dose reduction upon reinitiation. Some patients who experience Grade 3 adverse events may not resume ibrutinib treatment at all. As a result, the higher reporting rate of serious adverse events in the high-dose (standard-dose) group may largely reflect cautious prescribing practices in clinical settings. Patients at high risk of recurrent serious adverse events are less likely to restart the drug, which may lead to a lower proportion of serious adverse event reports in the reduced-dose group. In cases where the dose is gradually reduced from the standard level, it becomes difficult to accurately assess dose dependence from a pharmacological perspective.

Lastly, it is necessary to consider adjusting for differences in height, weight, and metabolic function that may affect the drug dose. There are also examples where SDR scores are calculated by adjusting each factor as a covariate (Hatano et al., 2023; Gu and Samarneh, 2025). In general cohort studies, covariate adjustment is a powerful tool for getting closer to the truth. However, spontaneous report databases have many points to note when adjusting for covariates (Noguchi et al., 2025). In this paper, we will omit statistical explanations (please refer to Ref. 21 for statistical details), but as mentioned above, spontaneous report databases are affected by reporting bias and have a lot of missing data. This is not limited to dosage. For example, in FAERS, gender is missing in approximately 13% of reports (Noguchi et al., 2021a). Due to the nature of spontaneous reporting, missing data is unlikely to be evenly distributed between genders. A similar problem may exist for age data. In FAERS, the annual proportion of missing age data increased from 21.9% in 2002 to 43.8% in 2018 (Pham et al., 2021). With these considerations, the validity of covariate adjustment may be compromised depending on the circumstances of missing data necessary for the analysis (e.g., height, weight, and presence of renal or hepatic dysfunction).

## 3 Alternative approaches and practical guidance for evaluating dose-dependence

To address this issue, alternative approaches should be considered. Integrating spontaneous reporting data with external sources such as prescription or dispensing databases can offer valuable context regarding background dose exposure patterns. For example, Mokbel et al. demonstrated that combining pharmacovigilance data with prescribing information enhances the interpretability of safety signals, even when individual-level dose information is lacking (Mokbel et al., 2022).

Moreover, more rigorous analytical methods—such as stratified analyses within observational cohorts or the use of marginal structural models—can help control for confounding and support stronger causal inference. The use of electronic health records (EHRs) or insurance claims databases has also been recommended for evaluating dose–response relationships (Gagne et al., 2012).

In summary, disproportionality analysis alone is insufficient for a reliable assessment of dose–response relationships. The integration of complementary data sources and robust methodologies is essential to draw valid and clinically meaningful conclusions.

## 4 Conclusion

In recent years, the ease of access to spontaneous reporting databases and calculating disproportionality scores have led to an increasing number of studies attempting to evaluate dose-dependence using disproportionality analysis. However, this trend is concerning, as the careless or overconfident use of disproportionality analysis for assessing dose-dependence—without a thorough understanding of its limitations—can lead to misleading conclusions. Even if the results appear to support previous findings, they are unlikely to be accepted unless the analysis is conducted with proper awareness of the limitations inherent in spontaneous reporting data. The five key points presented in this paper must be understood and properly analyzed.

## Abbreviations

ADR, adverse drug reaction; AF, atrial fibrillation; BCPNN, Bayesian Confidence Propagation Neural Network; EBGM, Empirical Bayesian Geometric Mean; EMA, European Medicines Agency; FAERS, FDA Adverse Event Reporting System; FDA, Food and Drug Administration; IRAF, ibrutinib-related atrial fibrillation, JADER; Japanese Adverse Drug Event Report database, PMDA; Pharmaceuticals and Medical Devices Agency, PRR; Proportional Reporting Ratio, READUS-PV; REporting of A Disproportionality analysis for drUg Safety signal detection using spontaneously reported adverse events in PharmacoVigilance, ROR; Reporting Odds Ratio, SDR; Signals of Disproportionate Reporting.
